# IQGAP1 and IQGAP2 are Reciprocally Altered in Hepatocellular Carcinoma

**DOI:** 10.1186/1471-230X-10-125

**Published:** 2010-10-26

**Authors:** Colin D White, Hema Khurana, Dmitri V Gnatenko, Zhigang Li, Robert D Odze, David B Sacks, Valentina A Schmidt

**Affiliations:** 1 Department of Pathology, Brigham and Women's Hospital and Harvard Medical School, Boston, MA 02115, USA; 2 Department of Medicine, Stony Brook University, Stony Brook, NY 11794, USA

## Abstract

**Background:**

IQGAP1 and IQGAP2 are homologous members of the IQGAP family of scaffold proteins. Accumulating evidence implicates IQGAPs in tumorigenesis. We recently reported that IQGAP2 deficiency leads to the development of hepatocellular carcinoma (HCC) in mice. In the current study we extend these findings, and investigate IQGAP1 and IQGAP2 expression in human HCC.

**Methods:**

IQGAP1 and IQGAP2 protein expression was assessed by Western blotting and immunohistochemistry. IQGAP mRNA was measured by quantitative RT-PCR. The methylation status of the *Iqgap2 *promoter was determined by pyrosequencing of bisulfite-treated genomic DNA.

**Results:**

IQGAP1 and IQGAP2 expression was reciprocally altered in 6/6 liver cancer cell lines. Similarly, immunohistochemical staining of 82 HCC samples showed that IQGAP2 protein expression was reduced in 64/82 (78.0%), while IQGAP1 was present in 69/82 (84.1%). No IQGAP1 staining was detected in 23/28 (82.1%) normal livers, 4/4 (100.0%) hepatic adenomas and 23/23 (100.0%) cirrhosis cases, while IQGAP2 was increased in 22/28 (78.6%), 4/4 (100.0%) and 23/23 (100.0%), respectively. Although the *Iqgap2 *promoter was not hypermethylated in HCC at any of the 25 CpG sites studied (N = 17), IQGAP2 mRNA levels were significantly lower in HCC specimens (N = 23) than normal livers (N = 6).

**Conclusions:**

We conclude that increased IQGAP1 and/or decreased IQGAP2 contribute to the pathogenesis of human HCC. Furthermore, downregulation of IQGAP2 in HCC occurs independently of hypermethylation of the *Iqgap2 *promoter. Immunostaining of IQGAP1 and IQGAP2 may aid in the diagnosis of HCC, and their pharmacologic modulation may represent a novel therapeutic strategy for the treatment of liver cancer.

## Background

Hepatocellular carcinoma (HCC) is the fifth most common cancer and the third most common cause of cancer-related death in the world [1]. It accounts for over 80% of all human liver cancer, and is responsible for between 500,000 and 1 million worldwide deaths annually [2]. Predisposing factors for HCC include chronic hepatitis B and C virus infections (HBV and HCV, respectively), exposure to aflatoxin B1, chronic alcohol consumption, or any hepatic disease associated with cirrhosis. Nevertheless, the molecular pathogenesis of HCC remains largely unknown. Recognized abnormalities in HCC include aberrant signaling through the mitogen-activated protein kinase, PI3K/Akt and mTOR pathways, and overactivation of several growth factor receptors (although research has focused mainly on the epidermal growth factor receptor) [3]. Recurrent allelic losses or gains have also been detected on 14 chromosome arms in more than 30% of all HCCs analyzed [4]. Despite the large number of scientific and clinical studies performed to date, overall survival of patients with HCC has not improved in the last two decades.

There are three IQGAP proteins in humans, termed IQGAP1, IQGAP2 and IQGAP3 [5]. IQGAP1 is the best-characterized member of the IQGAP family. Unlike IQGAP2, which is expressed primarily in the liver and platelets [6,7], and IQGAP3, where expression is limited to the brain [8], IQGAP1 is expressed ubiquitously [5]. IQGAP1 binds F-actin through calponin homology domains [9], interacts with multiple calmodulin molecules (in a Ca^2+^-regulated fashion) through repetitive IQ motifs (IQxxxRGxxR), and binds the Rho GTPases Cdc42 and Rac1 by means of a C-terminal RasGAP-related domain [5]. In addition to the established binding partners listed above, IQGAP1 associates with the ERK and MEK kinases [10,11], β-catenin [12,13], E-cadherin [14,15], adenomatous polyposis coli (APC) [16], mTOR [17], and Sec 3 and 8 (which are involved in exocytosis and invasion) [18]. IQGAP1 has been shown to regulate cell proliferation and migration *in vitro *[19-21], and is overexpressed in aggressive cancers [22]. In order to elucidate the physiological functions of IQGAP2 (one of the less well studied IQGAP1 homologs), a conventional *Iqgap2 *knockout mouse was generated in our laboratory [6]. We showed that IQGAP2 deficiency results in an 86% incidence of HCC. Of equal importance, mice deficient in both *Iqgap1 *and *Iqgap2 *(*Iqgap1*^*-/-*^*/Iqgap2*^*-/-*^) display relative protection against HCC, and have improved long-term survival. These data suggest that, at least in mice, changes in IQGAP expression contribute to the pathogenesis of HCC.

In humans, *Iqgap2 *silencing, by hypermethylation, contributes to the pathogenesis of certain forms of gastrointestinal cancer [23]. For example, *Iqgap2 *methylation was detected in 47% of gastrointestinal tumors, but not in normal mucosa. Additionally, IQGAP2 protein was absent from all samples in which the *Iqgap2 *promoter was hypermethylated, and a significant correlation was noted between *Iqgap2 *methylation and cancer aggressiveness. These data, viewed in conjunction with the data from our mouse model, prompted us to hypothesize that decreased IQGAP2 expression, as a result of hypermethylation of the *Iqgap2 *promoter, may contribute to the pathogenesis of human HCC. In the present study, our aim was to examine IQGAP1 and IQGAP2 expression in human HCC, their sensitivity and specificity as biomarkers of this type of tumor, and the methylation profile of the *Iqgap2 *promoter.

## Methods

### Cell Culture and Western Blotting

All cells were maintained in Dulbecco's Modified Eagles Medium (DMEM) supplemented with 10% (v/v) fetal bovine serum and 1% (v/v) penicillin/streptomycin. For IQGAP1 and IQGAP2 expression analyses, cell monolayers were placed on ice, washed twice with ice-cold phosphate-buffered saline (PBS), and lysed in lysis buffer (50 mM Tris (pH 8.0), 100 mM NaF, 30 mM Na_4_P_2_O_7_, 2 mM Na_2_MoO_4_, 5 mM EDTA, 2 mM Na_3_VO_4_) supplemented with 10 μg/ml aprotinin, 10 μg/ml leupeptin and 1 mM phenylmethylsulfonyl fluoride. Clarified cell lysates were equalized for protein concentration using the modified Bradford Assay (Bio-Rad Laboratories, Hercules, CA), resolved by SDS-PAGE, and processed by Western blotting. IQGAP1 and IQGAP2 expression was measured by Western blotting cell lysates with rabbit polyclonal anti-IQGAP1 (generated and characterized previously in our laboratory [24]; dilution 1:1,000) or mouse monoclonal anti-IQGAP2 (Upstate Biotechnology, Lake Placid, NY; clone BB9; dilution 1:1,000) antibodies, respectively. All blots were also probed with mouse monoclonal anti-β-Tubulin (Cell Signaling Technology, Danvers, MA; dilution 1:2,000) antibody to verify protein loading.

### Study Groups

A total of 66 biopsy (N = 26) and surgical resection (N = 40) routinely processed formalin-fixed paraffin-embedded (FFPE) specimens, from 66 patients (M/F ratio; 46/20, median age; 60.5 years), were randomly selected from the tissue archives of the Department of Pathology, Brigham and Women's Hospital, between the years 2005 and 2009. These included 30 HCC specimens, 4 hepatic adenomas, 23 cases of cirrhosis and 9 normal livers. In addition, a tissue microarray (TMA) (US Biomax, Rockville, MD) containing 52 HCC specimens and 19 normal livers (M/F ratio; 62/9, median age; 51 years) was also included in the study (Table 1). Hematoxylin and eosin stained sections were reviewed independently by two pathologists for confirmation of the diagnoses and grading of the tumors (according to International Union against Cancer (UICC) guidelines). This study was approved by the Institutional Review Board of Brigham and Women's Hospital.

**Table 1:** Patient demographics and pathologic information.

Feature	BWH (%)	TMA (%)	Total (%)
*Sex*			
Male	46/66 (69.7)	62/71 (87.3)	108/137 (78.9)
Female	20/66 (30.3)	9/71 (12.7)	29/137 (21.1)
*Median Age (range)*	60.5 (30-86)	51 (19-78)	56 (19-86)
*Histopathological Diagnosis*			
Normal	9/66 (13.6)	19/71 (26.8)	28/137 (20.4)
Adenoma	4/66 (6.1)	0/71 (0.0)	4/137 (2.9)
Cirrhosis	23/66 (34.8)	0/71 (0.0)	23/137 (16.8)
Carcinoma	30/66 (45.5)	52/71 (73.2)	82/137 (59.9)
*Hepatocellular Carcinoma Grade*			
Well Differentiated	8/30 (26.7)	11/52 (21.1)	19/82 (23.2)
Moderately Differentiated	13/30 (43.3)	25/52 (48.1)	38/82 (46.3)
Poorly Differentiated	9/30 (30.0)	16/52 (30.8)	25/82 (30.5)

BWH, Brigham and Women's Hospital; TMA, tissue microarray

### Immunohistochemistry

**Figure 1:**
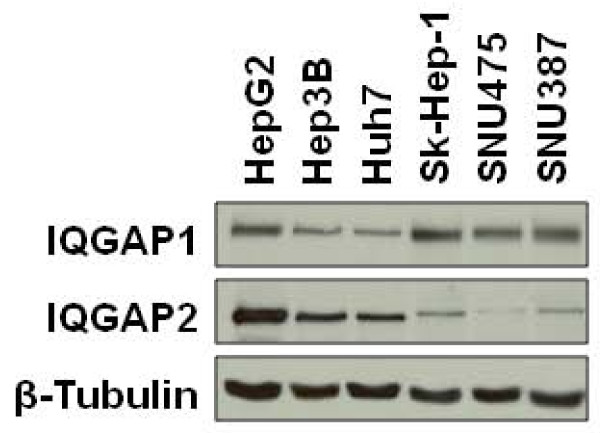
**IQGAP1 and IQGAP2 are reciprocally altered in human liver cancer cell lines**. Equal amounts of cell lysate from the cultured liver cancer cell lines indicated were resolved by SDS-PAGE and processed by Western blotting. Immunoblots were probed with anti-IQGAP1, anti-IQGAP2 and anti-β-Tubulin antibodies. Representative data from four independent experiments are shown.

FFPE blocks from Brigham and Women's Hospital were cut into 5 μm thick tissue sections and slides prepared using standard techniques. Mounted tissue sections were baked at 60°C for 20 min, deparaffinized in xylene and rehydrated through graded alcohols. Antigens were retrieved by heating in 1 μM sodium citrate (pH 6.0) in a pressure cooker at 125°C for 30 s. Non-specific staining was blocked using Dako Protein Block (Dako, Carpinteria, CA) according to the manufacturer's instructions. Anti-IQGAP1 (dilution 1:2,000) and anti-IQGAP2 (dilution 1:100) primary antibodies were diluted in Dako Antibody Diluent and incubated with the tissue sections for 1 h at room temperature. Staining was visualized using Dako Envision and developed with a DAB Chromogen substrate. Immediately after visualization, sections were dipped in DAB Enhancer, counterstained with hematoxylin, dehydrated through graded alcohols and xylene, and mounted. Appropriate positive and negative controls were used throughout all staining and interpretation. Antibody specificity for each IQGAP has previously been validated using protein from *Iqgap1*^*-/- *^and *Iqgap2*^*-/- *^mice [6]. Nevertheless, we confirmed these results using a panel of human HCC cell lines (Figure 1). Those cells with higher levels of IQGAP1 had lower levels of IQGAP2, and those with higher levels of IQGAP2 had lower levels of IQGAP1 (Figure 1). These data strongly suggest that each IQGAP antibody is highly specific for its respective protein.

### Immunostaining Interpretation

IQGAP1 and IQGAP2 immunostaining was blindly evaluated by two independent pathologists with a high degree of interobserver agreement (>90%). Staining for each antibody was considered positive if more than 10% of cells stained strongly in the cytoplasm.

### Quantitative real-time PCR

A commercial qRT-PCR TissueScan Array (OriGene, Rockville, MD) containing 23 individual cDNAs from patients with primary HCC of different stages, and 6 individual cDNAs from normal livers (M/F ratio; 19/10, median age; 63 years), was used for quantification of IQGAP1 and IQGAP2 mRNA transcripts. Each cDNA was normalized by the manufacturer against β-actin by RT-PCR. Oligonucleotide primer pairs were generated using Primer3 software (http://www.genome.wi.mit.edu), and designed to amplify ~200 bp PCR products at an annealing temperature of 71°C. Primer sequences were as follows: IQGAP1, forward: 5'-TCCAATAAGATGTTTCTGGGAGAT-3'; reverse: 5'-GATGATTTCACCAATGGAAATGTA-3'. IQGAP2, forward: 5'-GATGTAGGCATTTTCGATGTAAGA-3'; reverse: 5'-ATTTCTGTAGGCACTTCACTTTCC-3'. PCR reactions were initiated at 95°C for 15 s and cycled 39 times at 94°C for 30 s, 55°C for 30 s and 72°C for 30 s. mRNA was quantified by monitoring real-time fluorimetric intensity of SYBR green I at a reading temperature of 70°C using a 7300 thermocycler (Applied Biosystems, Foster City, CA). Relative mRNA abundance was determined using the comparative threshold cycle number (Δ-Ct method) [25], and normalized to the cDNA amount as previously described [26].

### Promoter Methylation Assay by Pyrosequencing

#### Genomic DNA isolation from FFPE liver samples

Tissue sections (3 per preparation) were deparaffinized in xylene for 5 min, rehydrated through graded alcohols and digested using 200 μg/ml proteinase K at 54°C overnight. Formalin cross-linking was reversed by incubation at 90°C for 1 h, and genomic DNA was isolated using the QIAamp DNA FFPE Tissue kit (Qiagen, Valencia, CA) according to the manufacturer's instructions.

#### Bisulfite treatment of genomic DNA

Bisulfite conversion of unmethylated CpG islands was completed using the EZ DNA Methylation Gold kit (Zymo Research, Orange, CA) according to the manufacturer's instructions. Bisulfite treatment converts all non-methylated cytosines (C) into thymidines (T), whereas all methylated Cs remain. Briefly, 1-2 μg of genomic DNA was treated with CT conversion reagent for 10 min at 98°C and 190 min at 64°C. After conversion, DNA was loaded onto a Zymo Spin IC column, washed with M-Wash buffer, and treated with M-Desulphonation buffer for 20 min at room temperature. Bisulfite-treated DNA was eluted from the column in 12 μl of M-Elution buffer, and the resulting DNA yield was evaluated using a 2100 Bioanalyzer (Agilent Technologies, Santa Clara, CA).

#### Assay design and PCR

The pyrosequencing technique allows quantitative determination of methylation at selected CpG sites in the gene promoter region [27]. A genomic DNA sequence corresponding to the human *Iqgap2 *promoter region (Chromosome 5:75734588 - 75735487) was downloaded from the UCSC Genome browser (http://genome.ucsc.edu/), and a text editor used to model bisulfite conversion of methylated CpG sites. The resulting modified DNA sequence was imported into Assay Design software (version 1.0.6) and used to generate primers for pyrosequencing. In parallel, primers for PCR amplification of the *Iqgap2 *promoter region were designed using the internet-based BiSearch primer design algorithm for bisulfite-converted DNA sequences (http://bisearch.enzim.hu). This algorithm allows searching of the entire bisulfite-converted human genome to identify primer pairs that exclude non-specific amplification (which is especially important for amplification of highly redundant promoter sequences) [28]. Primer sequences are listed in Additional File 1, and their location in the *Iqgap2 *promoter is shown in Additional File 2. Since the amount of DNA isolated from FFPE sections was insufficient to obtain a good quality product in one PCR reaction, two-round PCR amplification was used. For the first round, F1 and R1 were used as forward and reverse primers, respectively (Additional File 2). For the second round, F1 and Bio-R2 were used as forward and biotinylated-reverse primers, respectively (Additional File 2). As the GC-content of bisulfite-treated DNA was substantially altered, gradient PCR was used to optimize the annealing temperature (T between 50-60°C). An annealing temperature of 57°C resulted in the highest yield of PCR product, and no contaminating non-specific products were detected. PCR amplification was carried out according to the Qiagen protocol. Briefly, 10 μl of bisulfite-treated DNA was mixed with 10 μl of 2 × HotStarTaq Plus Master Mix. Primers were added at a final concentration of 0.5 μM in a reaction volume of 21 μl. PCR reactions were initiated at 95°C for 5 min, cycled 40 times at 94°C for 15 s, 57°C for 1 min and 65°C for 1 min, and completed with a final elongation step of 65°C for 10 min. PCR products were examined by electrophoresis on a 1.5% agarose gel in order to confirm the specificity of amplification. For the second round of PCR amplification, samples were diluted 100 times in TE Buffer (pH 7.5) and 2 μl was used per reaction. Second round PCR amplification was carried out as above.

#### Pyrosequencing

The 150 bp *Iqgap2 *promoter region was divided into three separate areas and three different sequencing primers, S1, S2 and S3, were used to sequence them (Additional File 1 and Additional File 2). The reaction was carried out in 96 well plates according to the manufacturer's instructions. Briefly, a biotin-labeled PCR product was captured on Streptavidin Sepharose High Performance beads (Amersham Biosciences, Uppsala, Sweden) in 80 μl of binding buffer. Beads containing the immobilized PCR product were washed with 70% ethanol, and the PCR product was denatured in 0.2 M NaOH. Pyrosequencing primer (0.3 μM) was annealed to the purified single-stranded PCR product at 80°C, and pyrosequencing was performed using a PyroMark MD pyrosequencer (Qiagen, Valencia, CA). The degree of methylation for each CpG site was expressed as a percentage of methylated cytosines over the sum of total cytosines. Non-CpG cytosine residues were used as built-in controls to verify bisulfite conversion. Genomic DNA from the Kato III human gastric cancer cell line (ATCC, Rockville, MD), which is known to have a hypermethylated *Iqgap2 *promoter [23], was used as a positive control in each experiment. Each assay also contained controls for self-annealing of sequencing primers and for self-annealing of a single-stranded PCR product.

### Statistical Analysis

The two-tailed Student's *t *test was used to analyze data from qRT-PCR experiments. For methylation studies, statistical significance was determined by analysis of variance (ANOVA), while correlation coefficients were established using regression analysis. For all comparisons, a *p *value < 0.05 was used to establish statistical significance. Box-and-whisker plots were used to display 5 descriptive statistics: the median, the lower and upper quartiles, and the minimum and maximum data values. The bottom and top of the box represent the 25^th ^and the 75^th ^percentiles (the lower and upper quartiles, respectively), and the band near the middle of the box represents the median (the 50^th ^percentile). Whiskers (error bars) below and above the box indicate the 10th and 90th percentiles (shown only for plots with N = 9 or more). Symbols outside whiskers represent outliers.

## Results

### IQGAP1 and IQGAP2 are Reciprocally Altered in Human Liver Cancer Cell Lines

We determined the relative amounts of IQGAP1 and IQGAP2 in a panel of human liver cancer cell lines. Equal amounts of cell lysate were processed by Western blotting. As anticipated, IQGAP levels varied among the cell lines. Those cells with higher levels of IQGAP1 have lower levels of IQGAP2, and those with higher levels of IQGAP2 have lower levels of IQGAP1 (Figure 1). In particular, Sk-Hep-1, SNU475 and SNU387 cells had high IQGAP1 and low IQGAP2 expression (Figure 1).

### IQGAP1 Protein is Upregulated and IQGAP2 Protein is Downregulated in Human HCC

**Figure 2:**
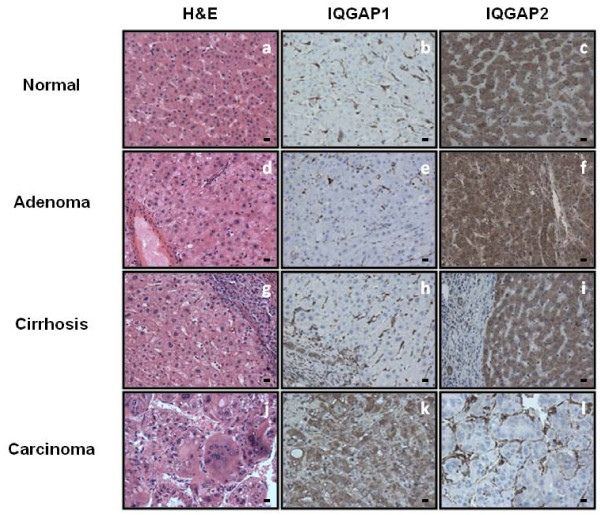
**IQGAP1 and IQGAP2 protein expression is altered in hepatocellular carcinoma**. Representative immunohistochemistry images showing IQGAP1 and IQGAP2 protein expression in normal (b-c), adenoma (e-f), cirrhosis (h-i) and carcinoma (k-l). Hematoxylin and eosin (H&E) stained images corresponding to each case are also shown (a, d, g, j). Scale bar represents 10 μm, and the final magnification is 400×.

The immunohistochemistry (IHC) data are summarized in Table 2. By IHC, IQGAP1 was detected in the hepatocytes of 5/28 (17.9%) normal livers, 0/4 (0.0%) hepatic adenomas and 0/23 (0.0%) cirrhosis cases (Figure 2 and Table 2). In contrast, the expression of IQGAP1 was diffusely positive (>10% of tumor cells) in 69/82 (84.1%) HCC specimens (Figure 2 and Table 2) (30/30 samples from Brigham and Women's Hospital and 39/52 samples from the TMA). The expression of IQGAP2 was diffusely positive in the hepatocytes of 22/28 (78.6%) normal livers, 4/4 (100.0%) hepatic adenomas and 23/23 (100.0%) cirrhosis cases (Figure 2 and Table 2). However, only 18/82 (22.0%) HCC specimens were positive for IQGAP2 (Figure 2 and Table 2) (2/30 samples from Brigham and Women's Hospital and 16/52 samples from the TMA). Of note, only 3 of 11 (27.3%) HCC specimens retrieved from and stained at the tissue archives of the Department of Pathology, Stony Brook University, were positive for IQGAP1 and negative for IQGAP2 (data not shown). To maintain consistency in IHC staining and interpretation, these samples have been excluded from our analysis. Although unlikely (as our results are consistent between the HCC specimens from Brigham and Women's Hospital and those from the TMA), we cannot rule out the possibility of differences in tissue preparation processes at different institutions accounting for these discrepant data. In all cases, the sinusoidal lining cells also showed strong IQGAP1 and IQGAP2 immunoreactivity.

**Table 2:** Immunohistochemistry results

Diagnosis	IQGAP1 (% Positive)	IQGAP2 (% Positive)
Normal	5/28 (17.9)	22/28 (78.6)
Adenoma	0/4 (0.0)	4/4 (100.0)
Cirrhosis	0/23 (0.0)	23/23 (100.0)
Carcinoma	69/82 (84.1)	18/82 (22.0)

### IQGAP2 mRNA is Downregulated in Human HCC

**Figure 3:**
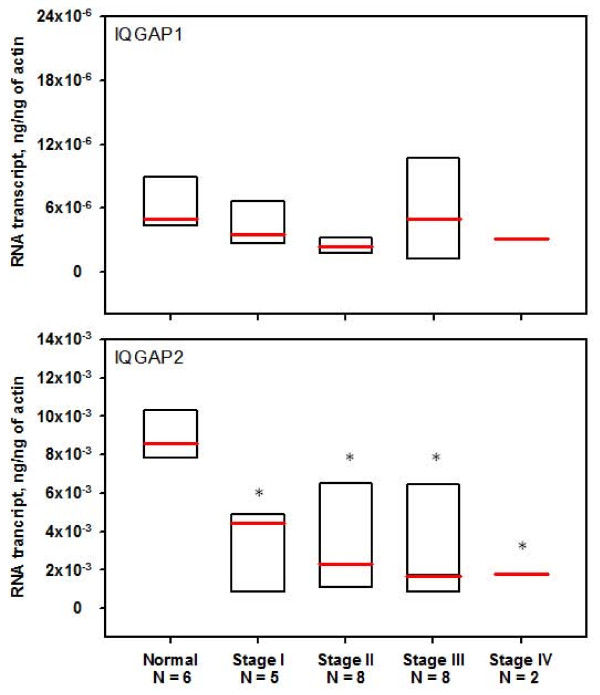
**IQGAP2 RNA transcript expression is altered in hepatocellular carcinoma**. IQGAP1 (top panel) and IQGAP2 (bottom panel) mRNA expression in cDNAs from patients with HCC and normal livers was evaluated by qRT-PCR. The boxed area represents 50% of samples (from the 25^th ^to the 75^th ^percentile) and the band inside the box represents the median. *, *p *< 0.05 versus normal livers.

mRNA expression analyses did not detect a significant alteration of IQGAP1 expression in HCC at any stage, nor was there a difference between IQGAP1 mRNA levels in cancer and normal tissue (Figure 3). In contrast, expression of the IQGAP2 transcript in HCC specimens was significantly decreased (*p *< 0.05) compared to normal livers (Figure 3), and the magnitude of this decrease was progressive between normal tissue and stage II HCC. No further statistically significant reduction was evident at stages III and IV. Interestingly, we found that normal livers have significantly (~1000-fold) more IQGAP2 mRNA than that of IQGAP1 (Figure 3).

### The Iqgap2 Promoter is not Hypermethylated in HCC

**Figure 4:**
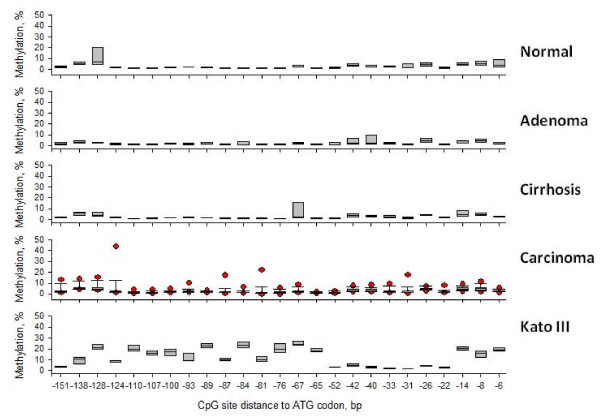
**The *Iqgap2 *promoter is not hypermethylated in hepatocellular carcinoma**. The degree of methylation for each CpG site was expressed as a percentage of methylated cytosines over the sum of total cytosines. The data are shown for 25 CpG sites and grouped by tissue type. Genomic DNA from the Kato III human gastric cancer cell line, which is known to have high methylation levels of the *Iqgap2 *gene promoter [23], was used as a positive control. The boxed area represents 50% of samples (from the 25^th ^to the 75^th ^percentile) and the band inside the box represents the median. Error bars (whiskers) indicate the 10th and 90th percentiles (shown only for plots with N = 9 or more). Symbols outside whiskers represent outliers.

To determine if IQGAP2 downregulation in HCC was due to hypermethylation of the *Iqgap2 *gene promoter, we performed pyrosequencing of bisulfite-treated genomic DNA from patient tissues. Efficient genomic DNA extraction requires a sufficient amount of FFPE tissue. Therefore, only the surgically resected HCC specimens, and not the biopsy or TMA specimens, were analyzed. 17 HCC specimens, 3 hepatic adenomas, 8 cirrhosis cases and 6 normal livers were used for methylation analysis. The overall methylation level of the *Iqgap2 *promoter was 3.1% in HCC specimens and 3.3% in normal livers (Figure 4 and Additional File 3). The hepatic adenomas and cirrhosis cases had *Iqgap2 *methylation levels comparable to that of normal livers (2.3% and 3.3%, respectively). To exclude the possibility that low methylation levels detected in FFPE samples may be due to damage of the genomic DNA by formalin, 2 snap-frozen HCC specimens and 3 normal livers from the National Disease Research Interchange (Philadelphia, PA) were also analyzed. In all cases, the results obtained were similar to those from FFPE tissue (Additional File 3).

## Discussion

We recently showed that IQGAP1 and IQGAP2 have opposing roles in a murine model of hepatic carcinogenesis [6]. In that study, *Iqgap2*^*-/- *^mice developed age-dependent HCC, whereas mice deficient in both *Iqgap1 *and *Iqgap2 *displayed relative protection against HCC and showed significantly improved long-term survival. These data suggest that, in HCC, IQGAP2 may represent a tumor suppressor and IQGAP1 an oncogene. In the current study, we evaluated the expression of IQGAP1 and IQGAP2 in human HCC. We showed that a reciprocal relationship existed between IQGAP1 and IQGAP2 expression in human liver cancer cell lines. Furthermore, IQGAP2 was downregulated in 78.0% of HCC specimens, and IQGAP1 protein was overexpressed in 84.1% of tumors. Finally, we demonstrated that IQGAP2 mRNA is decreased in HCC compared to normal livers (although we did not detect any significant change in the IQGAP1 transcript), and showed that the *Iqgap2 *promoter is not hypermethylated in HCC. Viewed collectively, these data strongly suggest that IQGAP1 and IQGAP2 contribute to the pathogenesis of HCC, and that these proteins are highly sensitive and specific biomarkers of this type of tumor.

Our data indicate that Sk-Hep-1, SNU475 and SNU387 cells have high levels of IQGAP1 and low levels of IQGAP2. Interestingly, these cells are mesenchymal and lack E-cadherin, while the other cell lines we studied have E-cadherin and are epithelial [29]. Loss of E-cadherin results in metastasis and a poor prognosis in many tumors [30]. Congruent with this, microarray analysis revealed that genes involved in invasion and metastasis are overexpressed in Sk-Hep-1, SNU475 and SNU387 cells, but not in HepG2, Hep3B or Huh7 cells [31] (which have low IQGAP1 and high IQGAP2 expression). Viewed collectively, these data suggest that increased IQGAP1 and/or decreased IQGAP2 expression may be a characteristic of a more invasive and metastatic HCC phenotype. Nevertheless, as we did not observe a difference in IQGAP1 positivity or IQGAP2 negativity between different HCC grades, it is also possible that these observations are a consequence of cell line immortalization. Future studies are necessary to reconcile these discrepant data.

Differentiation of HCC from benign hepatocellular tumors, such as hepatic adenomas, macroregenerative nodules, and high grade dysplastic nodules, can be difficult morphologically [32,33]. Several immunohistochemical markers, such as CD10, polyclonal CEA, and Hep Par1 may be used to help determine a hepatocellular origin of a particular lesion, but are not helpful in differentiating benign from malignant tumors [34,35]. Moreover, Glypican-3, a heparin-sulfate proteoglycan recently reported to show a high degree of specificity for HCC versus benign hepatocellular proliferations, is limited by its relatively low sensitivity [33,36]. In the current study, we demonstrated a high degree of sensitivity and specificity for IQGAP1 positivity, and IQGAP2 negativity, in HCC. Indeed, 84.1% of HCCs were positive for IQGAP1, whereas all hepatic adenomas, cirrhosis cases, and most (82.1%) normal livers were IQGAP1 negative. Similarly, IQGAP2 was negative in 78.0% of HCCs, but was positive in 100%, 100% and 78.6% of hepatic adenomas, cirrhosis cases and normal livers, respectively. Based on these data, we propose that IQGAP1 and IQGAP2, either alone or in combination, are highly sensitive and specific biomarkers of HCC. As a result, their use may be valuable in routine diagnostic pathology.

As mentioned above, our qRT-PCR results showed that IQGAP2 transcript expression in human HCC was significantly lower than normal tissue. To our knowledge, we are the first group to report this finding in human HCC. The only other report of reduced IQGAP2 mRNA in HCC comes from our previous study in mice [6]. Consistent with our current findings, IHC revealed that IQGAP2 protein, which was abundant in normal livers, was undetectable in most (78.0%) HCC specimens studied. Our qRT-PCR results also indicated that IQGAP1 mRNA expression did not differ significantly between normal livers and HCC. These findings are different from both our current IHC data, and mRNA data observed by other investigators [37]. Several factors may account for these differences, including the source of the original samples. For instance, our qRT-PCR experiments were performed on a commercial cDNA array for which no information regarding the etiology of HCC was available. Furthermore, it has been demonstrated that the expression of certain genes in HCC may differ depending on a patient's HBV and HCV status [38,39]. Moreover, although we could not detect IQGAP1 protein in normal hepatocytes, it was expressed in Kupffer cells. Thus, if the cDNA in the array specimens was crudely extracted from homogenized liver, rather than from hepatocytes exclusively, possible changes in IQGAP1 mRNA expression may have been masked by mRNA from other cell types. Recent examination of microarray datasets of human HCC revealed that the *Iqgap1 *gene is significantly upregulated in human HCC specimens compared to normal livers [37]. Conversely, in agreement with our results, Liao and colleagues reported no significant difference in *Iqgap1 *expression between normal livers and HCC [40]. A more quantitative assessment of *Iqgap1 *gene expression in a larger cohort of HCC specimens is necessary to reconcile these discrepancies.

Silencing of tumor suppressor genes by promoter hypermethylation is the most extensively studied epigenetic mechanism in tumorigenesis [41-43]. In our current study, we hypothesized that downregulation of IQGAP2 expression in HCC specimens may be due to aberrant methylation of the *Iqgap2 *promoter. To test this theory, we utilized pyrosequencing of bisulfite-treated DNA, and evaluated the *Iqgap2 *promoter methylation profile of our HCC specimens. Pyrosequencing is a quantitative technique which allows for precise determination of methylation of individual CpG sites within a specific CpG island. Additionally, it has been shown to be effective for methylation analysis of FFPE tissues [44]. Other commonly used methylation analysis methods, such as methylation specific PCR, do not allow resolution of single-CpG sites (which is especially important for the analysis of genes with heterogeneous methylation patterns). A methylation level of ≤ 5% is considered background noise, and thus has questionable significance [45]. The overall *Iqgap2 *methylation levels detected in our current study were less than 5% in both FFPE HCC specimens and in normal livers. In contrast, *Iqgap2 *hypermethylation was detected in Kato III cells, suggesting that our pyrosequencing assay was technically sound. We confirmed the results of our FFPE HCC specimens and normal livers using an independent set of snap-frozen patient tissue. Viewed collectively, these data strongly suggest that methylation of the *Iqgap2 *promoter is not the principle mechanism by which IQGAP2 is downregulated in HCC. It has been reported that ectopic expression of specific miRNAs in HCC cells results in silencing of *Iqgap1 *and a concomitant decrease in cell proliferation [46]. Thus, it is possible that IQGAP2 expression in HCC may also be regulated by miRNAs, instead of by promoter methylation.

This study is the first assessment of IQGAP1 and IQGAP2 expression in human HCC. Based on the data presented here, the stochiometry between IQGAP1 and IQGAP2 is central to hepatocellular carcinogenesis. The precise mechanism by which IQGAPs contribute to neoplastic transformation and tumor progression is still poorly understood. Numerous IQGAP1 binding partners are known to be involved in tumorigenesis [22]. In contrast, little is known about the proteins with which IQGAP2 interacts. Future studies will provide insight into the role of IQGAPs in liver cancer.

## Conclusions

We have shown that IQGAP2 expression is downregulated in more invasive and metastatic liver cancer cell lines and most human HCC tissue. Furthermore, our data indicate that this downregulation is not a result of hypermethylation of the *Iqgap2 *promoter. In contrast, IQGAP1 is overexpressed in more aggressive liver cancer cell lines and the majority of HCC specimens. While future studies will address the mechanism underlying this reciprocal change, these findings validate the relevance of the *Iqgap2*^-/- ^mouse model to human disease. Immunostaining of IQGAP1 and IQGAP2 may aid in the diagnosis of HCC, and their pharmacologic modulation may represent a novel therapeutic strategy for the treatment of liver cancer.

## List of Abbreviations

ANOVA: analysis of variance; APC: adenomatous polyposis coli; DMEM: Dulbecco's Modified Eagles Medium; FFPE: formalin-fixed paraffin-embedded; HBV: hepatitis B virus; HCC: hepatocellular carcinoma; HCV: hepatitis C virus; IHC: immunohistochemistry; PBS: phosphate-buffered saline; TMA: tissue microarray; UICC: International Union against Cancer.

## Competing interests

The authors declare that they have no competing interests.

## Authors' contributions

CDW, HK and RDO performed and interpreted all immunohistochemistry. DVG performed and interpreted all qRT-PCR and methylation analyses. ZL performed and interpreted all Western blotting. DBS provided intellectual input into many experiments. VAS performed and interpreted all methylation analyses and was in overall charge of the study. All authors have read and approved the final manuscript.

## Pre-publication history

The pre-publication history for this paper can be accessed here:

http://www.biomedcentral.com/1471-230X/10/125/prepub

## Supplementary Material

Additional file 1 **Oligonucleotide primers used for methylation analysis**. Click here for file

Additional file 2 **Design of the methylation assay**. The *Iqgap2 *promoter region, CpG sites and oligonucleotide primer positions are shown. Click here for file

Additional file 3 *Iqgap2 *promoter methylation summary. Click here for file
