# Supplementary material for: IQGAP1 and IQGAP2 are Reciprocally Altered in Hepatocellular Carcinoma
Source: BMC Gastroenterol. 2010 Oct 26;10:125. doi: 10.1186/1471-230X-10-125 (PMC2988069; doi:10.1186/1471-230X-10-125)
Supplement: Additional file 1 — Oligonucleotide primers used for methylation analysis. [file 1471-230X-10-125-S1.DOC]

| Primer Use | Primer Sequence and Orientation | Primer Description | Primer Name |
| --- | --- | --- | --- |
| PCR amplification of *Iqgap2* promoter region from bisulfite-treated DNA | 5’- GAGGAGAGTTTATTTTTATTTTAGTG -3’ | Forward | F1 |
|  | 5’- ATTACTACCTTCCAATTTCC -3’ | Reverse | R1 |
| Pyrosequencing | 5’- Biotin-CCCCACCCACTTACAACCATA -3’ | Biotinylated Reverse Primer | Bio-R2 |
|  | 5’- TGGTTTTGGAGAGAGAGTA -3’ | Sequencing primer 1 | S1 |
|  | 5’- GGAGGTTAGGGGAAAT -3’ | Sequencing primer 2 | S2 |
|  | 5’- GTAGAGTTCGCGAGTTTGGTTAG -3’ | Sequencing primer 3 | S3 |
