# Supplementary figures and images for: IQGAP1 and IQGAP2 are Reciprocally Altered in Hepatocellular Carcinoma
Source: BMC Gastroenterol. 2010 Oct 26;10:125. doi: 10.1186/1471-230X-10-125 (PMC2988069; doi:10.1186/1471-230X-10-125)

## Slide 1
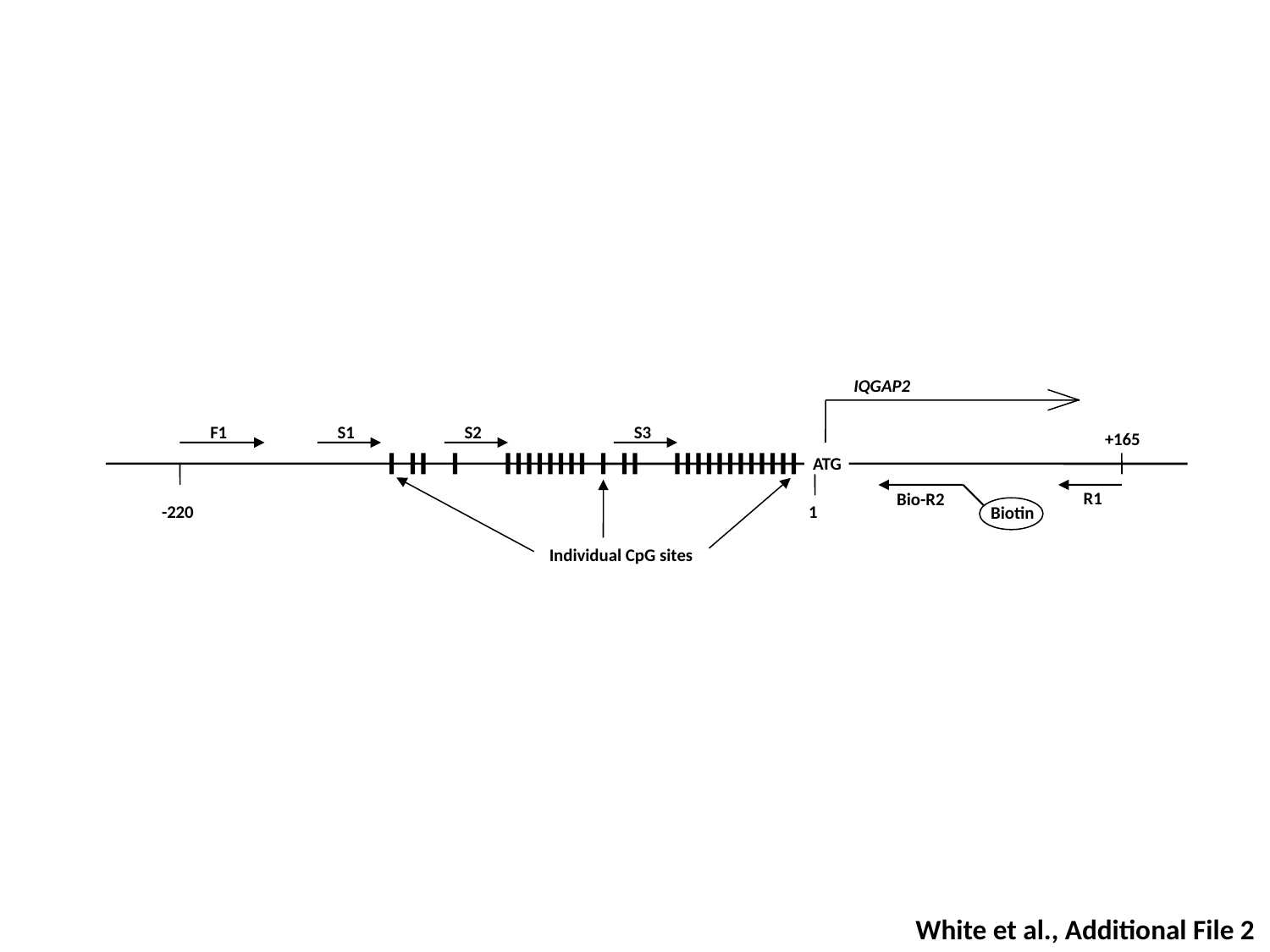

IQGAP2
F1
S1
S2
S3
+165
ATG
R1
Bio-R2
-220
1
Biotin
Individual CpG sites
White et al., Additional File 2

Supplement: Additional file 2 — Design of the methylation assay. The Iqgap2 promoter region, CpG sites and oligonucleotide primer positions are shown. [file 1471-230X-10-125-S2.PPTX]
